# Exploring the prediction performance for breast cancer risk based on volumetric mammographic density at different thresholds

**DOI:** 10.1186/s13058-018-0979-x

**Published:** 2018-06-08

**Authors:** Chao Wang, Adam R. Brentnall, Jack Cuzick, Elaine F. Harkness, D. Gareth Evans, Susan Astley

**Affiliations:** 1 Centre for Cancer Prevention, Wolfson Institute of Preventive Medicine, Queen Mary University of London, Charterhouse Square, London, EC1M 6BQ UK; 2 Centre for Imaging Science, School of Health Sciences, University of Manchester, Stopford Building, Oxford Road, Manchester, M13 9PT UK; 3 Department of Genomic Medicine, University of Manchester, St Mary’s Hospital, M13 9WL, Manchester, UK

**Keywords:** Breast density, Thresholding, Digital mammogram, Risk prediction, Breast cancer

## Abstract

**Background:**

The percentage of mammographic dense tissue (PD) defined by pixel value threshold is a well-established risk factor for breast cancer. Recently there has been some evidence to suggest that an increased threshold based on visual assessment could improve risk prediction. It is unknown, however, whether this also applies to volumetric density using digital raw mammograms.

**Method:**

Two case-control studies nested within a screening cohort (ages of participants 46–73 years) from Manchester UK were used. In the first study (317 cases and 947 controls) cases were detected at the first screen; whereas in the second study (318 cases and 935 controls), cases were diagnosed after the initial mammogram. Volpara software was used to estimate dense tissue height at each pixel point, and from these, volumetric and area-based PD were computed at a range of thresholds. Volumetric and area-based PDs were evaluated using conditional logistic regression, and their predictive ability was assessed using the Akaike information criterion (AIC) and matched concordance index (mC).

**Results:**

The best performing volumetric PD was based on a threshold of 5 mm of dense tissue height (which we refer to as VPD5), and the best areal PD was at a threshold level of 6 mm (which we refer to as APD6), using pooled data and in both studies separately. VPD5 showed a modest improvement in prediction performance compared to the original volumetric PD by Volpara with ΔAIC = 5.90 for the pooled data. APD6, on the other hand, shows much stronger evidence for better prediction performance, with ΔAIC = 14.52 for the pooled data, and mC increased slightly from 0.567 to 0.577.

**Conclusion:**

These results suggest that imposing a 5 mm threshold on dense tissue height for volumetric PD could result in better prediction of cancer risk. There is stronger evidence that area-based density with a 6 mm threshold gives better prediction than the original volumetric density metric.

## Background

The percentage of mammographic density (PD) that appears white in a mammogram and reflects the relative amount of fibroglandular tissue in the breast is a well-established risk factor for breast cancer [1]. PD is the most predictive marker of breast cancer for women after familial causes and polygenic markers when adjusted for age and body mass index (BMI) [2]. For area-based PD, fibroglandular and fatty tissues may be segmented by thresholding, and this is usually achieved by a semi-automatic approach where the threshold is chosen by the investigator using software such as Cumulus [3]. There has been recent evidence that increasing the conventional brightness threshold might better predict breast cancer risk: this has been demonstrated in Korean women with “for presentation” (processed) full-field digital mammograms [4, 5], and Australian women with digitised film mammograms [6].

In addition to subjective visual assessment, another approach for PD estimation using digital mammograms is volumetric density measurement via a fully automated system. Commercial volumetric PD systems including Volpara [7] and Quantra [8] have shown good agreement with semi-automated thresholding and an association with risk of breast cancer [9]. In Volpara, pixel values are calibrated so that the height (amount) of dense tissue at any given point in a mammogram can be estimated, and based on these heights and the estimated breast volume, volumetric density can be determined. By default all dense tissue, regardless of the height at any pixel position, is included to compute the dense volume. However, there appear to be no published studies that have looked at whether applying a threshold to dense tissue heights, effectively excluding some less dense tissue as well as possibly thin sheets or strands of tissue that have similar attenuation coefficients to glandular tissue, could result in better prediction of breast cancer risk.

The aim of this paper is to investigate whether volumetric or area-based PD can be adjusted by varying dense tissue height thresholds so as to better predict breast cancer risk. In previous research [4–6] thresholding was based on pixel brightness from visual assessment, whereas here thresholds on dense tissue heights from volumetric density estimation are used. This allows the calculation of breast density and the application of a chosen threshold to be fully automated (i.e. without manual visual assessment) on digital mammograms. In addition, our thresholding analysis is based on Western women with digital raw mammograms, and to our knowledge this has not been previously examined. An important benefit of using raw images compared to processed images is that it could reduce the discrepancies between different machines due to manufacturers’ proprietary processing algorithms.

## Methods

### Setting and study design

Two case-control studies were designed as a part of the Predicting Risk Of breast Cancer At Screening (PROCAS) cohort, in Manchester, UK [10]. The first case-control study had 317 cases and 947 controls while the second had 318 cases and 935 controls. A detailed description of the data in the two studies has been reported previously [11, 12] (the sample used for analysis differs slightly; see Appendix). Briefly, in the first case-control study, cases comprised women with cancer detected at first screen on entry into the PROCAS cohort, and we refer to this dataset as study 1. As in our previous study [11], the craniocaudal (CC) views of the contralateral breast for cases and the left breast for controls were used. In the second case-control study, each woman had a normal screening mammogram (no cancer detected) on entry into the PROCAS cohort, but an interval or screen-detected cancer arose subsequently, and we refer to this dataset as study 2. Similar to our previous study [11], the CC views of the contralateral breast for cases and the same side for controls were used. The mammograms were obtained on average three years prior to diagnosis of breast cancer and from the same cohort as study 1. In both studies women were matched approximately 3:1 (controls vs cases) by age, BMI, hormone replacement therapy (HRT) use and menopausal status.

### Mammograms

All digital raw (“for processing”) mammograms were acquired using a GE Senographe system. Volumetric density, especially the height of dense tissue at each point in the mammogram, was assessed using Volpara 1.5.2 (Volpara Health Technologies, Wellington, New Zealand).

### Density measurements

One output from the Volpara software is a “density map” - it contains data on dense tissue height at every point in the mammogram, based on an analysis of pixel values and imaging parameters. Whilst no thresholding is applied in the default output of the software, different threshold values can be tested such that only densities with a height greater than a certain threshold value are included for computing total dense volume. For instance, when a threshold level of 5 mm is used, only those density heights greater than 5 mm are employed to calculate the total dense volume. We refer to this approach to computing PD as volumetric PD (VPD) in this paper, and specifically the default volumetric PD output by Volpara as VPD0 (i.e. the threshold level is 0 mm).

The aforementined approach focuses on percentage of volumetric density as the end point. An alternative approach is to look at the two-dimensional area of dense tissue within the breast: here this is defined as the number of pixels with dense tissue heights greater than a chosen threshold. This is then divided by the total number of pixels in the breast and expressed as a percentage area of dense tissue. As with the volumetric approach, a series of threshold values can be considered. We refer to this as areal PD (APD) in this paper. Note that although APD is an areal measurement, the underlying basis is still volumetric density because dense tissue height (or effectively volume) at each point in the mammogram was used.

### Statistical analysis

PDs at various threshold levels, ranging from 0 to 25 mm, were evaluated using conditional logistic regression, based on the pooled data (study 1 and 2 combined) and on study 1 and 2 separately. The Akaike information criterion (AIC) and matched concordance index (mC) [13] were calculated to measure prediction performance. AIC is a likelihood-based statistic derived from the information theory and is a well-established method for model comparison [14]. A lower AIC value indicates better model performance. mC is a modification of the concordance index (or area under the receiving operator characteristic curve, AUC) for matched case-control studies, and gives an average concordance index within matched groups. Bootstrap with 10,000 replications was used to assess whether the difference in mC from different models was statistically significant. All *p* values are two-sided.

Since biologic phenotypes between screen-detected and interval cancers are different, a further analysis was conducted to test whether there was any significant difference between screen-detected and interval breast cancers. In addition to the fixed threshold level for every woman, sensitivity analysis was conducted by varying the threshold according to a woman’s characteristics based on a linear model, using age, BMI, thickness and total volume of the breast to explore the difference between varying and fixed thresholds.

## Results

### Study characteristics

The demographic characteristics of the women in both studies are presented in Table 1. Age, BMI, menopausal status and HRT use were well-matched between cases and controls in both studies. The median 10-year Tyrer-Cuzick score was higher for cases than controls. The majority of women never used HRT, were postmenopausal, parous and ethnically white.

**Table 1:** Demographics of Study 1 (cancers detected at first screen on entry to the PROCAS study) and Study 2 (cancers detected at a subsequent screen or between screening rounds)

	Study 1			Study 2		
	Controls	Cases	*p* value	Controls	Cases	*p* value
	Number (%)	Number (%)		Number (%)	Number (%)	
Age at consent (years)			0.9997			0.9997
< 50	53 (6)	19 (6)		46 (5)	16 (5)	
50–54	242 (26)	79 (25)		194 (21)	64 (20)	
55–59	153 (16)	52 (16)		164 (18)	58 (18)	
60–64	229 (24)	77 (24)		286 (31)	96 (30)	
65–69	196 (21)	66 (21)		198 (21)	68 (21)	
70+	74 (8)	24 (8)		47 (5)	16 (5)	
HRT use			0.0778			0.9320
Unknown	14 (1)	9 (3)		23 (2)	6 (2)	
Never	568 (60)	208 (66)		475 (51)	166 (52)	
Previous	315 (33)	83 (26)		332 (36)	110 (35)	
Current	50 (5)	17 (5)		105 (11)	36 (11)	
BMI (kg/m^2^)			0.9954			0.9389
Unknown	1 (0)				1 (0)	
< 25	332 (35)	112 (35)		335 (36)	117 (37)	
25–29	331 (35)	111 (35)		341 (36)	113 (36)	
≥ 30	283 (30)	94 (30)		259 (28)	87 (27)	
Menopausal status			0.4272			0.9889
Unknown	31 (3)	10 (3)		32 (3)	12 (4)	
Premenopausal	92 (10)	32 (10)		67 (7)	22 (7)	
Perimenopausal	112 (12)	38 (12)		134 (14)	46 (14)	
Postmenopausal	712 (75)	237 (75)		702 (75)	238 (75)	
Ethnic origin			0.1880			0.2208
Other/unknown	52 (5)	24 (8)		81 (9)	35 (11)	
White	895 (95)	293 (92)		854 (91)	283 (89)	
Parity			0.7134			0.0399
Unknown	1 (0)			1 (0)	4 (1)	
Nulliparous	112 (12)	40 (13)		91 (10)	44 (14)	
Parous	834 (88)	277 (87)		843 (90)	270 (85)	
Tyrer-Cuzick (10 year risk, % (median, Q1–Q3))	2.74 (2.18–3.58)	2.94 (2.29–3.88)	0.0006	2.67 (2.09–3.55)	2.91 (2.24–4.05)	<.0001
Volumetric PD (median, Q1–Q3)	4.90 (3.63–7.19)	5.43 (4.06–8.13)	0.0034	4.79 (3.58–7.01)	5.51 (3.81–7.98)	0.0044

The *p* values, from likelihood-ratio chi-square tests, indicate whether there are significant difference between cases and controls

*HRT* hormone replacement therapy, *BMI* body mass index, *Q1* 25th percentile, *Q3* 75th percentile, *PD* percent density

### Results for pooled data

**Fig. 1:**
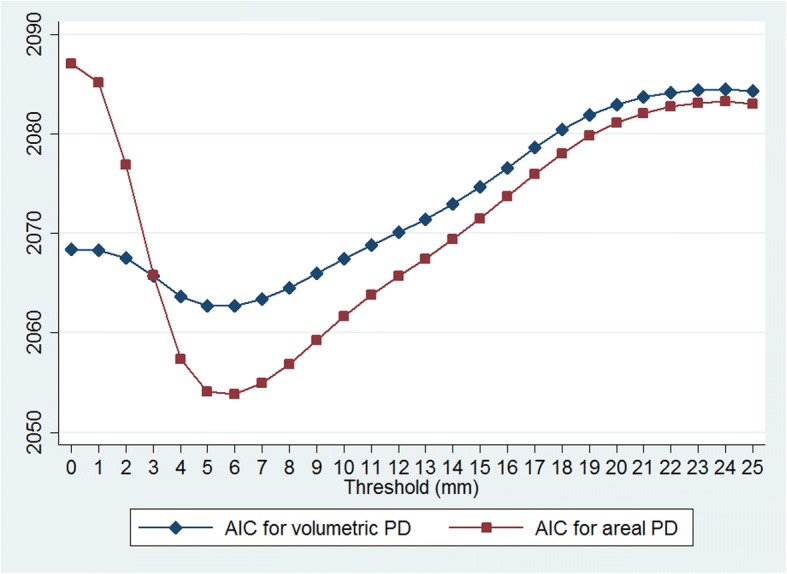
Akaike information criteria (AIC) using volumetric and areal percent density in pooled data

Conditional logistic regression was used to evaluate model fit at various threshold levels using both datasets combined. The resulting AICs for VPDs and APDs are presented in Fig. 1. It can be seen that both VPDs and APDs have their lowest value at the 5–6 mm threshold level, where improvement over original volumetric PD (i.e. VPD0) is clear. APD at the threshold of 6 mm achieved the lowest AIC overall.

**Fig. 2:**
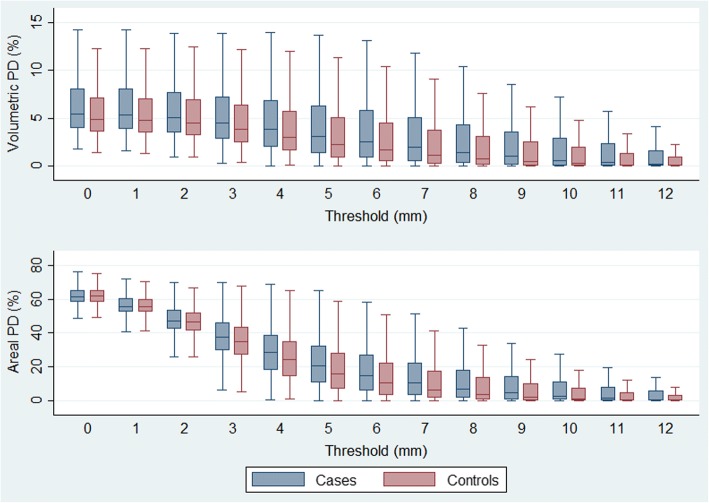
Distribution of volumetric percent density (VPD) and areal percent density (APD) at different thresholds

**Fig. 3:**
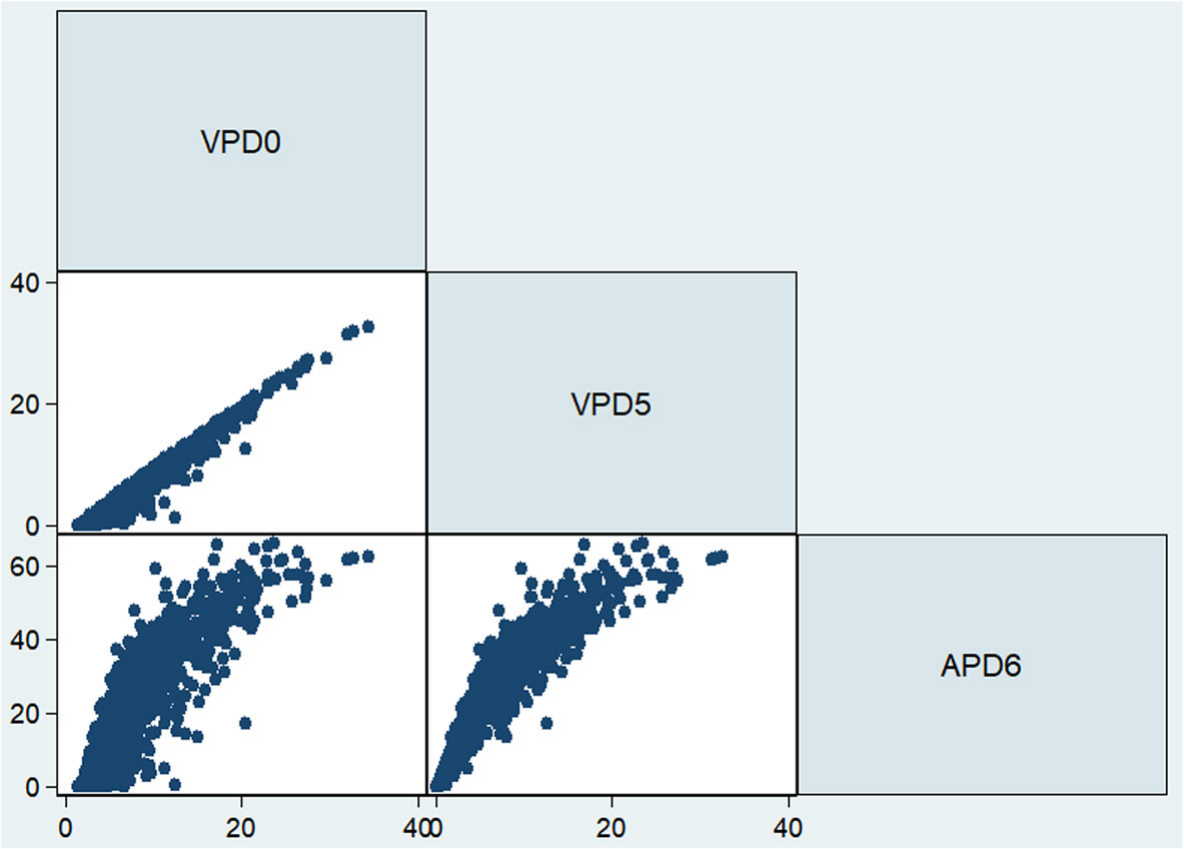
Correlation between volumetric percent density with a 0 mm threshold (VPD0), VPD with a 5 mm threshold (VPD5) and areal percent density with a 6 mm threshold (APD6)

Distributions of VPDs and APDs at different threshold levels (0–12 mm) were inspected using box plots as shown in Fig. 2. Correlations between VPD0 and the best performing VPD and APD - VPD5 and APD6, respectively, are presented in Fig. 3. The Spearman statistic was 0.95 for correlation between VPD0 and VPD5, 0.90 for correlation between VPD0 and APD6 and 0.98 for correlation between VPD5 and APD6.

Table 2 compares the results of five modelling schemes using different sets of risk predictors: (1) VPD0; (2) volumetric PD at 5 mm (VPD5); (3) areal PD at 6 mm (APD6); (4) VPD0 + VPD5 and (5) VPD0 + APD6. Each modelling scheme was denoted as M1 to M5, respectively. M1 represents the original volumetric PD estimated by Volpara (i.e. zero or no thresholding) and its model performance was used as the baseline for comparison with other models. M1 was then compared with M2 and M3 which were based on 5 mm and 6 mm thresholds for VPD and APD, respectively, as the best fit was found at these levels of threshold as shown above. M4 was used to explore whether the prediction performance for VPD5 can be further improved by adding the original Volpara estimate (VPD0); similarly, M5 was used to explore whether VPD0 adds information once having already controlled for APD6. The model with the lowest AIC indicates the best modelling approach for breast cancer risk prediction.

**Table 2:** Modelling results for the pooled data

	Standardised odds ratio (95% CI)				
	M1	M2	M3	M4	M5
Volumetric PD (0 mm)	1.26			0.46	0.90
	(1.15, 1.39)			(0.26, 0.82)	(0.74, 1.10)
Volumetric PD (5 mm)		1.29		2.72	
		(1.18, 1.42)		(1.56, 4.74)	
Areal PD (6 mm)			1.34		1.47
			(1.22, 1.47)		(1.21, 1.78)
Model fit statistics					
AIC	1727.15	1721.25	1712.63	1715.81	1713.50
mC	0.567	0.577	0.577	0.583	0.582
	(0.539, 0.596)	(0.548, 0.606)	(0.549, 0.605)	(0.555, 0.611)	(0.553, 0.610)
χ^2^	21.98	27.87	36.49	35.32	37.62

Standardized odds ratio is the change in odds for a standard deviation increase in predictors. Confidence intervals (CI) are presented in parentheses for the predictors in each model

*M* model, *PD* percent density, *AIC* Akaike information criterion, *mC* matched concordance index

**Fig. 4:**
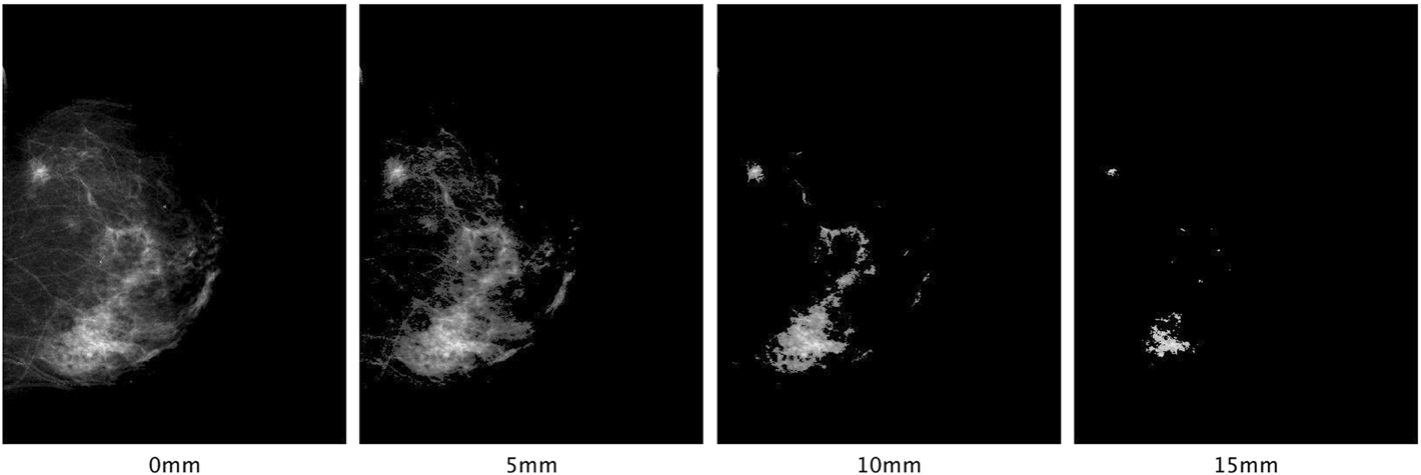
A visual comparison of “density map” using 0–15 mm threshold levels. Traditional volumetric density such as from the Volpara software uses a 0 mm threshold (no threshold). VPD0, volumetric percent density with a 0 mm threshold; VPD5, VPD with a 5 mm threshold; APD6, areal percent density with a 6 mm threshold

As seen in Table 2, M3, the model using only APD at 6 mm, was the best performing in terms of AIC. Compared to M1, the model using original volumetric PD (VPD0), the AIC was substantially improved with ΔAIC = 14.52. mC also increased slightly from M1 to M3 (from 0.567 to 0.577); whilst the change in mC was small it was still statistically significant (*p* value = 0.019). To show the effect of thresholding, an example is presented in Fig. 4, which shows thresholding of a mammogram at different levels.

### Results for study 1 and 2

Following analysis based on pooled data, a series of conditional logistic regression models for study 1 (cancers detected at the first screen on entry into the PROCAS cohort) and study 2 (cancers diagnosed subsequently) were explored, as well as screen-detected vs interval cancers within study 2. Similarly, five modelling schemes (M1–M5) were tested and the results are presented in Tables 3 and 4.

**Table 3:** Modelling results for study 1 in which cancers were detected at initial screening

	Standardised odds ratio (95% CI)				
	M1	M2	M3	M4	M5
Volumetric PD (0 mm)	1.25			0.51	0.96
	(1.10,1.43)			(0.24,1.09)	(0.73,1.25)
Volumetric PD (5 mm)		1.28		2.44	
		(1.13,1.46)		(1.16,5.14)	
Areal PD (6 mm)			1.31		1.36
			(1.15,1.50)		(1.05,1.78)
Model fit statistics					
AIC	867.80	865.01	862.55	863.87	864.45
mC	0.559	0.564	0.556	0.573	0.560
	(0.518, 0.599)	(0.524, 0.604)	(0.515, 0.595)	(0.533, 0.613)	(0.519, 0.600)
χ^2^	10.81	13.60	16.06	16.74	16.16

Standardized odds ratio is the change in odds for a standard deviation increase in predictors. Confidence intervals (CI) are presented in parentheses for the predictors in each model

*M* model, *PD* percent density, *AIC* Akaike information criterion, *mC* matched concordance index

**Table 4:** Modelling results for study 2 in which cancers were detected after the initial screening

	Standardised odds ratio (95% CI)				
	M1	M2	M3	M4	M5
Volumetric PD (0 mm)	1.27			0.42	0.84
	(1.11,1.47)			(0.18,0.97)	(0.63,1.12)
Volumetric PD (5 mm)		1.31		3.04	
		(1.14,1.50)		(1.32,6.97)	
Areal PD (6 mm)			1.37		1.58
			(1.19,1.57)		(1.20,2.08)
Model fit statistics					
AIC	861.35	858.24	851.99	855.87	852.61
mC	0.576	0.590	0.599	0.597	0.605
mC	(0.536, 0.616)	(0.551, 0.630)	(0.559, 0.640)	(0.557, 0.636)	(0.565, 0.644)
χ^2^	11.17	14.27	20.52	18.64	21.91

Standardized odds ratio is the change in odds for a standard deviation increase in predictors. Confidence intervals (CI) are presented in parentheses for the predictors in each model

*M* model, *PD* percent density, *AIC* Akaike information criterion, *mC* matched concordance index

As with the pooled data, M3, the model using only APD at 6 mm, was the preferred model in terms of AIC in study 1 (Table 3). Compared to M1, there was modest improvement in the AIC (ΔAIC = 5.25). Statistically, however, there was little difference in mC between M1 and M3 (*p* value = 0.60). Adding VPD0 to APD6 (M5) failed to improve model performance in terms of the AIC.

In study 2, M3 was again the best model in terms of the AIC (Table 4). Compared to VPD0 (M1), APD6 (M3) was considerably superior in terms of the AIC (ΔAIC = 9.36). mC for M3 was also significantly higher than for M1 (*p* value <0.001). VPD0 did not add statistically significant information after controlling for APD6 (M5 vs M3, *p* value = 0.24). Indeed, it can be shown that similar to the result shown in Fig. 1, APD6 (M3) was a better predictor than volumetric or other areal PDs at different thresholds both in studies 1 and 2.

A series of likelihood-ratio tests were performed on the aforementioned models to test whether there was any significant difference between screen-detected and interval cancers within study 2. The interaction term was found to be statistically significant for APD6 (M3, *p* value = 0.004; M5, *p* value = 0.003). Since VPD0 in M5 did not add information to APD6, the final model for prediction of screen-detected and interval cancers was based on APD at a threshold level of 6 mm (i.e. APD6 with additional interaction term). The resulting standardised odds ratio for APD at the 6 mm threshold was 1.81 for interval cancers (95% CI = 1.42–2.30) and 1.18 for screen-detected cancers (95% CI = 0.99–1.40).

## Discussion

This paper explores the impact of various levels of density thresholding on the performance in prediction of breast cancer using digital mammograms. To achieve this, a range of threshold levels from 0 to 25 mm were tested. For VPD, the threshold was varied so that only dense tissue where heights were greater than a given value were included to calculate the total dense volume of the breast. For APD, we counted the number of dense pixels above the threshold level and compared this with the total number of pixels in the breast to derive the areal PD.

Results from both case-control studies and from the pooled data confirm that a threshold level of 5 mm or 6 mm, either volumetric or areal, improves cancer risk prediction compared to original VPD without thresholding. However, the improvement with VPD at the higher thresholds was relatively small. This is not surprising given the strong correlation between VPD0 and VPD5 (spearman *ρ* approximately 0.95 in both studies). On the other hand, APD at threshold of 6 mm (APD6) achieved the best results across all models tested, including VPD and APD at various threshold levels, with ΔAIC = 14.52 for the pooled data compared to VPD0. It is worth noting that APD6 was also highly correlated with VPD0 (spearman *ρ* approximately 0.90 in both studies), which is not surprising given both APD and VPD measure relative dense tissue albeit from a different perspective. In addition to fixed threshold levels, varying threshold levels were also examined with the level of threshold based on a woman’s characteristics such as age, BMI and breast volume; however, the AIC did not improve, so a fixed threshold is preferred.

We also explored the impact of thresholding by visualising mammograms after areas with less dense tissue were excluded. As illustrated in Fig. 4, thresholding at 5 mm filtered out a large portion of lower-density areas, and was roughly comparable to *Altocumulus* presented by previous research [6]. Further thresholding at higher levels at 10 and 15 mm seems to exclude too much information, thus no further improvement in prediction was observed at these levels. It appears that by introducing a suitable threshold level (e.g. 5–6 mm), much of the “noise” presented in the mammograms (including fine structures with low attenuation) is removed and hence results in a more predictive PD estimate.

It is also interesting that whilst APD performed much worse than VPD initially when the level of thresholding was low, APD became better than VPD when a threshold level of 4 mm or above was applied, as shown in Fig. 1. This suggests that VPD is relatively insensitive to the “noise” presented in mammograms compared to APD, since VPD is essentially a weighted sum (i.e. if all dense tissue heights were the same then VPD would be equivalent to APD). However, after exclusion of the noise component, the weights (dense tissue heights) became less relevant, resulting in APD being a better predictor. This is interesting because it suggests that once the density at each point in the mammogram reaches some threshold, the measures are equally informative in terms of cancer risk despite local differences in density.

In terms of the biological plausibility for these findings, the major component of dense breast tissue is stroma [15], and pathways for breast cancer risk associated with dense tissue are likely to involve the stromal cells, extracellular matrix proteins and the epithelial component. It has also been shown that local density is associated with the location where cancer would develop [16]. However, the causal route between dense tissue and breast cancer is unknown, and research is ongoing in this important area [15]. For these reasons we do not speculate further on how this measure of breast density might better capture the biological mechanism for risk due to dense breast tissue. From a measurement accuracy point of view, however, an increased threshold may remove the areas of fat that look slightly grey on the image, which might reduce measurement error. Another possible explanation is that setting an appropriate threshold removes thin sheets or strands of tissue which have similar attenuation coefficients to glandular tissue, and exclusion of this type of tissue might contribute to better density estimation.

Consistent with previous studies [4–6], our results show that once the APD at the optimal threshold level is accounted for, conventional VPD0 no longer adds information - in fact models with multiple PD measurements (M4 and M5) performed worse than the model with only APD6 as a predictor (M3). While the standardised OR and mC, including those based on the original VPD estimated by Volpara (M1), might seem relatively low compared with some previous studies [6, 9], the results are broadly consistent with a body of previous research [4, 17, 18]. For example, Brandt et al. [17] compared VPD with BI-RADS using a large case-control sample (1911 cases and 4170 controls) and identified a similar discriminatory ability for Volpara VPD (AUC = 0.58, 95% CI 0.56–0.59) as in our study. It is also worth noting that the studies that have directly compared VPD by Volpara with established visual-based assessment such as BI-RADS and Cumulus have shown broadly similar ability for risk prediction [12, 17, 19], and so differences in predictive ability between studies might be due to other characteristics of the data. It is plausible that the predictive ability of a density measure differs across different sub groups of women and types of cancers, such as screen-detected and interval cancers as demonstrated here and by others [18]. This means the predictive ability likely depends on the composition of the study population, which may explain some of the differences between studies.

Previous studies have demonstrated that breast density adds accuracy to established breast cancer risk models such as the Tyrer-Cuzick and Gail models [20, 21], including in combination with single-nucleotide polymorphism risk panels [22]. It is therefore expected that this study will be of clinical importance, as an improved automated density measure is likely to help identify women who require additional screening and to help devise a risk-based screening/prevention strategy.

The strength of our approach, compared to previous studies [4–6], is that the process is fully automated without any human intervention. Also, by using raw (“for processing”) digital mammograms, differences due to manufacturers’ proprietary processing algorithms are reduced. Our approach, however, would benefit from testing in a wider range of settings. For example, the majority of women in our datasets were white and parous, so it would be important to validate our approach amongst other groups of women. Finally, the mammograms employed in our study are generated from a GE system. Nguyen et al. [5] found that prediction performance may vary considerably between different mammographic machines based on visual assessment. It would be interesting to further explore the impact of thresholding using different systems in which the image properties may differ, and how the method can be calibrated for mammograms from different systems and the resulting discriminatory power in different settings.

## Conclusion

This study examined volumetric and areal PDs defined by various thresholds, and found that APD at 6 mm is the best risk predictor of breast cancer in two case-control studies. The results presented in this study confirm findings from previous studies that dense tissue is more important for predicting breast cancer risk. Unlike previous studies where thresholding was based on pixel brightness by visual assessment, the approach adopted in this paper was based on the height of dense tissue calculated from volumetric density estimation, which enables our approach to be fully automated.

## Appendix

The number of cases and controls differs from a previous report that compared density measurements using the same women. The reasons are as follows. First, the first case-control study was a subset of one with 317 cases and 952 controls. Three women were excluded due to linkage errors between mammograms and questionnaire data. An additional two women were excluded because the CC view mammograms at the designated side of the breast (i.e. either left or right CC view) were unavailable.

The second case-control study originally had 338 cases and 1014 controls: 23 women were excluded because of unavailability of mammograms at the time of analysis (either no mammograms were provided for some women at the given side; or only mediolateral oblique (MLO) views were available but no CC views). A further 64 women were removed because the side of cancer (left or right) was unknown. A further 12 controls were removed during conditional logistic regression because they had no matched cases as a result of the aforementioned exclusions.

## Abbreviations

AIC: Akaike information criterion
APD: Areal percent density
AUC: Area under the receiver operating characteristic curve
BMI: Body mass index
CC: Craniocaudal
CI: Confidence interval
HRT: Hormone replacement therapy
mC: Matched concordance index
PD: Percent density
PROCAS: Predicting Risk Of breast Cancer At Screening
VPD: Volumetric percent density
